# Osteophytes, Enthesophytes, and High Bone Mass: A Bone-Forming Triad With Potential Relevance in Osteoarthritis

**DOI:** 10.1002/art.38729

**Published:** 2014-08-26

**Authors:** Sarah A Hardcastle, Paul Dieppe, Celia L Gregson, Nigel K Arden, Tim D Spector, Deborah J Hart, Mark H Edwards, Elaine M Dennison, Cyrus Cooper, Martin Williams, George Davey Smith, Jon H Tobias

**Affiliations:** 1 University of BristolBristol, UK; 2 University of Bristol, Bristol, UK, and University of Exeter Medical SchoolExeter, UK; 3 Oxford NIHR Musculoskeletal Biomedical Research Unit, University of Oxford, Oxford, UK, and University of SouthamptonSouthampton, UK; 4 King's College LondonLondon, UK; 5 University of SouthamptonSouthampton, UK; 6 Oxford NIHR Musculoskeletal Biomedical Research Unit, University of Oxford, Oxford, UK, and NIHR Nutrition Biomedical Research Centre, University of SouthamptonSouthampton, UK; 7 North Bristol NHS TrustBristol, UK

## Abstract

**Objective:**

Previous studies of skeletal remains have suggested that both enthesophytes and osteophytes are manifestations of an underlying bone-forming tendency. A greater prevalence of osteophytes has been observed among individuals with high bone mass (HBM) compared with controls. This study was undertaken to examine the possible interrelationships between bone mass, enthesophytes, and osteophytes in a population of individuals with extreme HBM.

**Methods:**

Cases of HBM (defined according to bone mineral density [BMD] Z scores on dual x-ray absorptiometry) from the UK-based HBM study were compared with a control group comprising unaffected family members and general population controls from the Chingford and Hertfordshire cohort studies. Pelvic radiographs from cases and controls were pooled and evaluated, in a blinded manner, by a single observer, who performed semiquantitative grading of the radiographs for the presence and severity of osteophytes and enthesophytes (score range 0–3 for each). Logistic regression analysis was used to identify significant associations, with a priori adjustment for age, sex, and body mass index.

**Results:**

In this study, 226 radiographs from HBM cases and 437 radiographs from control subjects were included. Enthesophytes (grade ≥1) and moderate enthesophytes (grade ≥2) were more prevalent in HBM cases compared with controls (adjusted odds ratio [OR] 3.00 [95% confidence interval (95% CI) 1.96–4.58], *P* < 0.001 for any enthesophyte; adjusted OR 4.33 [95% CI 2.67–7.02], *P* < 0.001 for moderate enthesophytes). In the combined population of cases and controls, the enthesophyte grade was positively associated with BMD at both the total hip and lumbar spine (adjusted *P* for trend < 0.001). In addition, a positive association between osteophytes and enthesophytes was observed; for each unit increase in enthesophyte grade, the odds of any osteophyte being present were increased >2-fold (*P* < 0.001).

**Conclusion:**

Strong interrelationships were observed between osteophytes, enthesophytes, and HBM, which may be helpful in defining a distinct subset of patients with osteoarthritis characterized by excess bone formation.

The term “enthesis” describes the site of insertion of a tendon, ligament, fascia, or articular capsule into bone (1,2). An enthesophyte is a bony spur arising at an enthesis, extending in the direction of pull of the ligament or tendon (3). Several conditions are associated with the formation of enthesophytes, including the seronegative spondyloarthritides, various endocrine disorders such as diabetes mellitus, local trauma, and calcium pyrophosphate deposition disease (1,4). However, enthesophytes may also be degenerative in nature (1) or may have no clear underlying cause (3,5).

Enthesophytes are a feature of diffuse idiopathic skeletal hyperostosis (DISH), a condition in which the presence of osteophytes around large joints has also been noted (1). This has led to speculation that the formation of osteophytes and enthesophytes may be manifestations of a common underlying process. In support of this concept, archaeologic studies involving direct examination of skeletons have revealed strong positive correlations between the presence of enthesophytes and the presence of osteophytes (3,6). Furthermore, in one study, an association between generalized enthesophyte formation and bony eburnation (sclerosis of bony surfaces thought to represent full-thickness cartilage loss) was seen (6), leading to the suggestion that osteoarthritis (OA) may represent a systemic disorder of bone in which the bony response to mechanical stress is abnormal. However, only a few studies have used joint imaging to examine the relationships between enthesophytes and osteophytes/OA, and conclusions have been inconsistent (7–9).

In contrast, an association between increased bone mineral density (BMD) and radiographic OA has been widely reported (10–12), and this association appears to be strongest with the bony features of OA, such as osteophytes (13). We recently carried out a study of radiographic hip OA in a population of individuals with high bone mass (HBM). In comparison with control subjects, HBM cases had an increased prevalence of OA, predominantly characterized by the presence of osteophytes and subchondral sclerosis, suggesting a propensity to form bone (14). Moreover, previous clinical phenotyping of these individuals showed that those with HBM more frequently had misshapen or extra bone, including at the tendon and ligament insertions, compared with a control population (15). This led us to speculate that enthesophytes may also form part of the HBM phenotype.

The aim of this study was to investigate the relationship between HBM and the presence of enthesophytes on pelvic radiographs. Specifically, we aimed to determine 1) whether HBM has an association with enthesophytes similar to that previously observed with osteophytes, and 2) whether osteophytes and enthesophytes are themselves associated within this population, and whether any observed relationship varies according to the presence or absence of HBM. We hypothesized that radiographic enthesophytes would be more prevalent in HBM cases, possibly reflecting a tendency to form excess bone, and that the presence and severity of osteophytes and enthesophytes would also be strongly associated.

## SUBJECTS AND METHODS

### HBM study population

The HBM study is a UK-based, multicenter observational study of adults with unexplained HBM, as fully described elsewhere (15). Briefly, 13 dual x-ray absorptiometry (DXA) databases in the UK were screened for T scores and/or Z scores for BMD greater than or equal to +4. All of the DXA images were inspected by trained clinicians for artefactual causes of elevated BMD on DXA; 49.4% of the scans were excluded because their high T scores and/or Z scores reflected degenerative disease/OA/scoliosis, and a further 15.5% of the scans were excluded for other reasons, including surgical/malignant/Pagetic artefacts (15). The definition of an HBM index case was refined to require either of the following criteria: 1) a Z score greater than or equal to +3.2 at the L1 vertebra of the lumbar spine plus a Z score greater than or equal to +1.2 at the total hip or 2) a Z score greater than or equal to +3.2 at the total hip plus a Z score greater than or equal to +1.2 at the L1 vertebra of the lumbar spine. Misclassification of HBM case status was minimized by using the Z score at L1, which, in contrast to the values in the lower lumbar spine, was previously found to be unassociated with lumbar spine OA as assessed on DXA images in a subgroup of HBM cases (15,16).

Index cases with unexplained HBM were recruited, and relatives and spouses of these individuals were invited to undergo DXA screening. Among first-degree relatives, HBM has been defined as a summed L1 Z score plus total hip Z score of greater than or equal to +3.2 (15). Applying this definition of HBM, 41% of the relatives screened were found to be affected, and were combined with the HBM index cases to form the HBM group. The remaining, unaffected first-degree relatives/spouses formed the family control group (15).

Cases and controls underwent identical assessments, including a structured interview and clinical examination. Anteroposterior (AP) pelvic radiographs and AP/lateral lumbar spine radiographs were obtained from participants ages ≥40 years. Written informed consent was obtained from all participants, consistent with the Declaration of Helsinki (17), and the study was approved by the Bath multicenter Research Ethics Committee (REC) and each National Health Service local REC. For this study, HBM cases were then divided into 5-year age bands by sex, prior to selection of additional controls by age- and sex-stratified random sampling from 2 population-based cohorts.

### General population controls

#### Chingford 1,000 Women Study controls

The Chingford 1,000 Women Study started in 1989 with an initial recruitment of 1,003 women ages 45–64 years from the age/sex register of a general practice in Chingford, North-East London (10); of these women, 470 (46.9%) had a radiographic followup evaluation at 20 years. Supine pelvic radiographs were obtained in followup years 2, 8, and 20; radiographs from year 20 were digital and those from years 2 and 8 were latterly digitized. Controls from the Chingford cohort were randomly selected in a 2:1 ratio of controls to female HBM cases within each age band, except the lower age band (ages 40–49 years) and upper age band (≥80 years), for which a control-to-case ratio of 3:1 was used. A single radiograph per participant was included; controls in the upper age bands were selected first, to ensure sufficient numbers of available radiographs.

#### Hertfordshire Cohort Study (HCS) controls

For the HCS cohort, ∼3,000 men and women born in Hertfordshire between 1931 and 1939 and still resident there in 1998–2003 were recruited (18). Recently, a subset of HCS participants were recruited into the European Project on Osteoarthritis (EPOSA) (19). As part of the EPOSA study, supine pelvic and/or knee radiographs were obtained from 207 men and 203 women (ages 71.5–80.6 years at the time of radiographic evaluation); these individuals formed the selection pool for the present study. The HCS EPOSA study controls were selected in a 2:1 ratio with HBM cases within each appropriate age band (ages 70–74 years, 75–79 years, and ≥80 years).

### Assessment of radiographs

#### Osteophytes

All case and control radiographs were pooled for assessment, with the files presented in a blinded random order. Radiographs were graded by a single observer (SAH) following focused radiologic training. Using an established atlas (20), the presence of osteophytes at each location within the hip joint (superior acetabular, medial femoral, and lateral femoral) was scored on a scale of 0–3. These scores were used to generate binary variables for the presence of any osteophyte (defined as any osteophyte score of ≥1), presence of moderate osteophyte (defined as any osteophyte score of ≥2), and presence of femoral osteophyte (defined as an osteophyte score of ≥1 in the medial or lateral femoral location) affecting either hip on each radiograph. Categorical variables for the maximum osteophyte grade per pelvis (scale 0–3), total number of osteophyte sites per pelvis (scale 0–6), and total osteophyte score per pelvis (scale 0–18) were also generated, with the latter score being obtained by summing all of the osteophyte grades at the 6 possible sites on each radiograph. The presence or absence of chondrocalcinosis was also noted (scale 0–1).

At the end of the study, 60 randomly selected radiographs were regraded by the primary observer and a second experienced observer (PD) to establish intra- and interrater reproducibility. Intrarater kappa values for the binary osteophyte variables at each hip joint were 0.73 for the presence of any osteophyte and 0.74 for moderate osteophyte, representing good intrarater agreement. However, interrater kappa values were substantially poorer, being 0.19 for the presence of any osteophyte and 0.33 for moderate osteophyte; this was mainly due to poor reproducibility of the scores for superior acetabular osteophytes between the 2 observers (as has been noted by other investigators [20]). Therefore, femoral osteophytes have been reported separately (interrater kappa value for the presence of binary femoral osteophyte 0.63).

#### Enthesophytes

**Figure 1:**
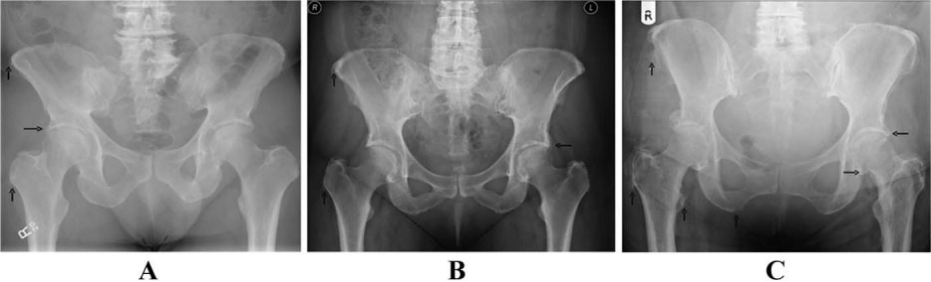
Pelvic radiographs obtained from our enthesophyte atlas, showing examples of mild enthesophytes (grade 1), characterized by subtle new bone formation at the anterior superior iliac spine (ASIS) and greater trochanter (vertical arrows) (A), moderate enthesophytes (grade 2), characterized by new bone formation mainly at the ASIS and greater trochanter (B), and florid enthesophytes (grade 3), characterized by marked new bone formation around the ASIS, iliac crests, greater and lesser trochanters, and, to a lesser extent, the ischial tuberosity (C). Horizontal arrows indicate the presence of osteophytes at the superior acetabular margin in A–C and at the left medial femur in C. Example images were made available as full-screen digital images to readers during scoring.

Enthesophytes were graded as 0 for absent, 1 for mild, 2 for moderate, or 3 for florid, based on the assessor's overall assessment of the entire radiograph, paying particular attention to the iliac crests, greater and lesser trochanters, and ischial tuberosities. To improve standardization, a consensus meeting was held prior to commencement of the study, in which 2–3 examples of each grade were identified and used to compile an atlas, which was then made available for reference. Atlas examples were selected from all of the pooled radiographs, following blinding of the case/control status. Examples of pelvic radiographs showing each enthesophyte grade are presented in Figure 1.

During the grading of enthesophytes, the assessor noted if the radiographic image was incomplete (missing ≥2 of the above-described sites), which would preclude accurate assessment; these radiographs were later excluded. The categorical enthesophyte grading (scale 0–3) was used to generate 2 binary variables for analysis: presence of any enthesophyte (grade ≥1) and presence of moderate enthesophytes (grade ≥2). Weighted intrarater and interrater kappa values for the categorical enthesophyte grade were 0.88 and 0.62, respectively, and intrarater and interrater kappa values for the binary variables were 0.80 and 0.55, respectively, for any enthesophyte and 0.92 and 0.50, respectively, for moderate enthesophytes.

### Assessment of covariates

Values for age (at the time of radiographic evaluations), sex, body mass index (BMI), and DXA-assessed BMD were obtained from the data sets of each study for use in the present analysis. BMI was calculated as weight (in kg) divided by height (in square meters), using the measurements obtained closest to the time of radiographic evaluation. Data on relevant self-reported medical conditions were available for the HBM study cases and controls, as were basic biochemical measures, including the levels of serum alkaline phosphatase and phosphate.

### Statistical analysis

Demographic statistics for the different study populations are summarized as the mean ± SD for continuous variables and counts (percentages) for categorical variables. In this case–control analysis, categorical variables were initially cross-tabulated, and percentages were calculated. The chi-square test was used to assess associations between binary variables. Logistic regression was used to examine associations between the exposure (HBM case status) and the binary enthesophyte/osteophyte outcome variables, with adjustment for a priori confounders (age, sex, and BMI). The odds ratios (ORs) with 95% confidence intervals (95% CIs) before and after adjustments are presented. Planned sensitivity analyses included 1) analyses that excluded HBM cases with any condition known to be associated with enthesophyte formation (1) or HBM cases with low serum alkaline phosphatase/phosphate levels, and 2) analyses that excluded HBM cases and family controls with evidence of possible DISH affecting the lumbar spine. Logistic regression was then used to examine associations between categorical enthesophyte/osteophyte grade and binary osteophyte/enthesophyte outcomes, with adjustment for confounders and stratification by HBM case status. Data were analyzed using Stata statistical software (release 12; StataCorp).

## RESULTS

### Selection and characteristics of the participants

**Figure 2:**
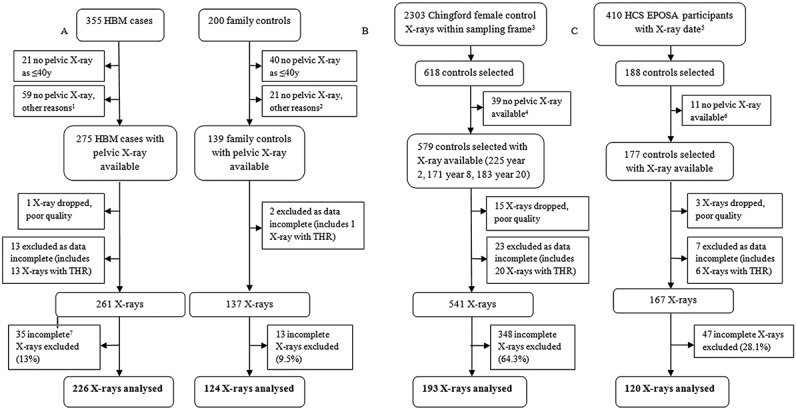
Selection of radiographs from high bone mass (HBM) cases (index plus affected relatives) and unaffected family controls (see ref.^15^ for a full description of the process of study recruitment) (A), radiographs from female control subjects in the Chingford 1,000 Women Study (B), and radiographs from male and female control subjects in the European Project on Osteoarthritis (EPOSA) substudy of the Hertfordshire Cohort Study (HCS) (C). ^1^Reason recorded for missing radiograph in HBM cases: unable to travel (n = 7), no radiographs at study center (n = 23), unable to attend/wait/comply (n = 4), patient declined (n = 8), not done (reason unknown) (n = 9), resides abroad (n = 2), or bilateral hip replacements (n = 6). ^2^Reason recorded for missing radiograph in family controls: did not continue in study (n = 1), unable to travel (n = 1), no radiographs at study center (n = 9), unable to attend/wait/comply (n = 2), subject declined (n = 4), not done (reason unknown) (n = 3), or bilateral hip replacements (n = 1). ^3^Sampling frame constructed from dates of the followup visits at years 2, 8, and 20. ^4^Reason recorded for missing radiograph in the Chingford Study controls: not found at time of request (n = 6), not digitized (n = 18), or unknown reason (n = 15). ^5^Sampling frame constructed from the EPOSA study radiograph appointment dates. ^6^Reason recorded for missing radiograph in the HCS EPOSA controls: unilateral hip radiograph only (n = 1), bilateral hip replacements (n = 3), or unknown (n = 7). ^7^Incomplete radiographs were defined as those missing ≥2 pelvic ligament insertion sites. THR = total hip replacement.

Figure 2 summarizes the selection of radiographs for inclusion. Those radiographs judged to be of unacceptable quality (1 case radiograph and 18 control radiographs) and those with missing osteophyte data (13 case radiographs and 32 control radiographs, including total hip replacement) were excluded from the outset. Incomplete radiographs were also excluded, particularly affecting earlier digitized radiographs from the Chingford cohort, in which the iliac crests were frequently not visible. In total, 226 HBM case radiographs and 437 control radiographs were included in the analysis.

Characteristics of the study population are shown in Table1. Controls tended to be older than cases (mean age 68.1 years versus 62.5 years). Cases had a higher mean BMI compared with controls (30.3 kg/m^2^ versus 27.7 kg/m^2^). Moreover, as expected, cases had higher BMD at both the total hip and the L1 vertebra. The BMD variables were approximately normally distributed, apart from 1 individual considered to be an extreme HBM outlier, with a total hip BMD of 2.47 gm/cm^2^. The proportion of women was approximately equal between the groups (76.6% of cases versus 78.5% of controls). Compared with the control subjects who were included, control subjects who were excluded (due to having incomplete radiographs) were younger, had a lower BMI, and were more often female (results not shown). The prevalence of enthesophytes and osteophytes was greater among cases compared with controls regardless of the binary cutoff chosen (Table1).

**Table 1:** Descriptive characteristics of the study population

		Controls			
	HBM cases (n = 226)	Family controls (n = 124)	Chingford controls (n = 193)	Hertfordshire controls (n = 120)	All controls (n = 437)
Age, mean ± SD years	62.5 ± 11.4	59.3 ± 12.9	69.4 ± 9.1	75.1 ± 2.7	68.1 ± 11.0
BMI, mean ± SD kg/m^2^*	30.3 ± 5.7	27.8 ± 4.7	27.8 ± 4.7	27.5 ± 3.9	27.7 ± 4.5
BMD, mean ± SD gm/cm^2^†					
Total hip	1.25 ± 0.17	0.97 ± 0.13	0.91 ± 0.12	0.94 ± 0.13	0.93 ± 0.13
L1 vertebra	1.40 ± 0.17	1.05 ± 0.16	0.89 ± 0.15	0.95 ± 0.19	0.95 ± 0.18
Female, no. (%)	173 (76.6)	60 (48.4)	193 (100.0)	90 (75.0)	343 (78.5)
Any enthesophytes, no. (%)	170 (75.2)	62 (50.0)	107 (55.4)	83 (69.2)	252 (57.7)
Moderate enthesophytes (grade ≥2), no. (%)	66 (29.2)	9 (7.3)	19 (9.8)	17 (14.2)	45 (10.3)
Any osteophyte, no. (%)	185 (81.9)	82 (66.1)	149 (77.2)	92 (76.7)	323 (73.9)
Moderate osteophyte (grade ≥2), no. (%)	69 (30.5)	18 (14.5)	37 (19.2)	23 (19.2)	78 (17.9)
Femoral osteophyte, no. (%)	65 (28.8)	21 (16.9)	44 (22.8)	34 (28.3)	99 (22.7)

* BMI = body mass index.

† Bone mineral density (BMD) variables were standardized according to the scanner type (Hologic for the Chingford and Hertfordshire study controls, mixed Lunar/Hologic for the high bone mass [HBM] cases and family controls depending on study center) using standard equations (see refs.^48^ and^49^). When the BMD values for both the right hip and the left hip were available (n = 73), the mean value was used. Sample sizes (no. of individuals or pelvises) for all variables are as shown, except for total hip BMD (n = 218 HBM cases, n = 123 family controls, n = 180 Chingford controls, and n = 120 Hertfordshire controls) and L1 BMD (n = 217 HBM cases, n = 123 family controls, n = 183 Chingford controls, and n = 120 Hertfordshire controls).

### Enthesophytes and osteophytes in HBM cases compared with controls

#### Regression analyses

Unadjusted regression analyses revealed an increased odds of the presence of any enthesophyte (OR 2.23 [95% CI 1.56–3.18], *P* < 0.001) and moderate enthesophytes (OR 3.59 [95% CI 2.36–5.48], *P* < 0.001) in HBM cases compared with controls (Table2). Similarly, the odds of any osteophyte being present (OR 1.59 [95% CI 1.07–2.38], *P* = 0.023), any moderate osteophyte being present (OR 2.02 [95% CI [1.39–2.94], *P* < 0.001), and any femoral osteophyte being present (OR 1.38 [95% CI 0.96–1.99], *P* = 0.085) were all increased in HBM cases compared with controls.

**Table 2:** Regression analysis of enthesophyte and osteophyte variables in HBM cases compared with all pooled controls*

Outcome	OR (95% CI), HBM cases vs. controls	*P*
Any enthesophyte		
Unadjusted model	2.23 (1.56–3.18)	<0.001
Adjusted model	3.00 (1.96–4.58)	<0.001
Moderate enthesophytes		
Unadjusted model	3.59 (2.36–5.48)	<0.001
Adjusted model	4.33 (2.67–7.02)	<0.001
Any osteophyte		
Unadjusted model	1.59 (1.07–2.38)	0.023
Adjusted model	2.24 (1.44–3.49)	<0.001
Moderate osteophyte		
Unadjusted model	2.02 (1.39–2.94)	<0.001
Adjusted model	2.32 (1.55–3.49)	<0.001
Any femoral osteophyte		
Unadjusted model	1.38 (0.96–1.99)	0.085
Adjusted model	1.67 (1.13–2.47)	0.011

* Osteophyte variables refer to the worse hip per pelvis. Values are the odds ratio (OR) with 95% confidence interval (95% CI) for each outcome in 226 high bone mass (HBM) cases and 437 pooled controls, in unadjusted regression analyses and in analyses adjusted for age, sex, and body mass index.

The presence of enthesophytes was positively associated with increasing age and male sex (results not shown). A positive association between enthesophytes and BMI was also observed. Therefore, regression analyses were rerun with adjustments for age, sex, and BMI, all of which strengthened the above-noted associations. The adjusted OR for any enthesophyte in HBM cases was 3.00 (95% CI 1.96–4.58; *P* < 0.001) (Table2), representing a 3-fold increased prevalence of enthesophytes in HBM cases compared with controls. Similarly, the adjusted OR for moderate enthesophytes was 4.33 (95% CI 2.67–7.02; *P* < 0.001). Furthermore, the associations between HBM case status and presence of osteophytes were also strong, although smaller in magnitude, with an ∼2-fold increase in the prevalence of any osteophyte and any moderate osteophyte in HBM cases compared with controls.

When we further adjusted the association between HBM case status and presence of any enthesophyte for the presence of osteophytes, only minimal attenuation of the association was observed (OR 2.74 [95% CI 1.78–4.21], *P* < 0.001). Similarly, no substantial attenuation was observed when the association between HBM case status and presence of any osteophyte was adjusted for the presence of enthesophytes (OR 1.95 [95% CI 1.24–3.07], *P* = 0.004), implying that osteophytes and enthesophytes are each independently associated with HBM (further details available from the corresponding author upon request).

#### Sensitivity analyses

##### Exclusion of individuals with comorbidities

Relevant comorbidities associated with enthesophyte formation present in HBM cases included diabetes (n = 26), psoriatic arthritis (n = 4), and hypoparathyroidism (n = 2). Excluding these individuals from the analysis did not alter the associations found between HBM case status and enthesophytes/osteophytes (details available from the corresponding author upon request). Six HBM cases with borderline–low serum phosphate levels (<0.7 mmoles/liter) were identified; excluding these cases from the analysis did not alter our findings (details available from the corresponding author upon request). Chondrocalcinosis within either hip joint was identified in 5.4% of the radiographs; excluding these radiographs did not materially affect the associations observed (results not shown). There were no cases of ankylosing spondylitis, hyperparathyroidism, acromegaly, or hypophosphatasia in the HBM group.

##### Exclusion of DISH cases

The flowing calcification and ossification typical of DISH (of which enthesophytes are a recognized feature), if present in the lumbar spine, could potentially lead to misclassification of HBM case status through artefactual elevation of the measured BMD (21,22). Therefore, we sought to establish what proportion of our study population with enthesophytes also had DISH affecting the spine, in particular the L1 vertebra used to define HBM. The widely used Resnick criteria for DISH (23) were originally applied to radiographs of the whole spine; however, in our study, digital spinal radiographs were available only for the HBM study cases and family controls and were obtained in the lumbar spine only.

The lumbar spine radiographs of all HBM cases and family controls with either florid or moderate (grade 2 or 3) enthesophytes (n = 75) were reviewed by the primary observer (SAH) along with a radiologist (MW), both of whom were blinded with regard to the case/control status. Definite or possible features of DISH affecting the L1 vertebra were observed in 19 individuals (18 HBM cases and 1 family control). Excluding these individuals resulted in slight attenuation of the OR for any enthesophyte in HBM cases compared with controls (OR 2.78 [95% CI 1.81–4.27], *P* < 0.001) and a more substantial attenuation of the OR for moderate enthesophytes (OR 3.10 [95% CI 1.86–5.18], *P* < 0.001). However, both associations remained strong, suggesting that the presence of DISH-related changes at L1 does not explain the HBM–enthesophyte association observed.

We further confirmed this finding by performing a sensitivity analysis that included only those HBM cases meeting the index case definition at the hip (total hip Z score greater than or equal to +3.2; n = 100). Strong associations persisted between HBM case status and the presence of both enthesophytes and osteophytes, when compared with the combined control group (details available from the corresponding author upon request).

### Analyses based on HBM cases and controls combined

#### Hip and L1 BMD according to enthesophyte grade

**Figure 3:**
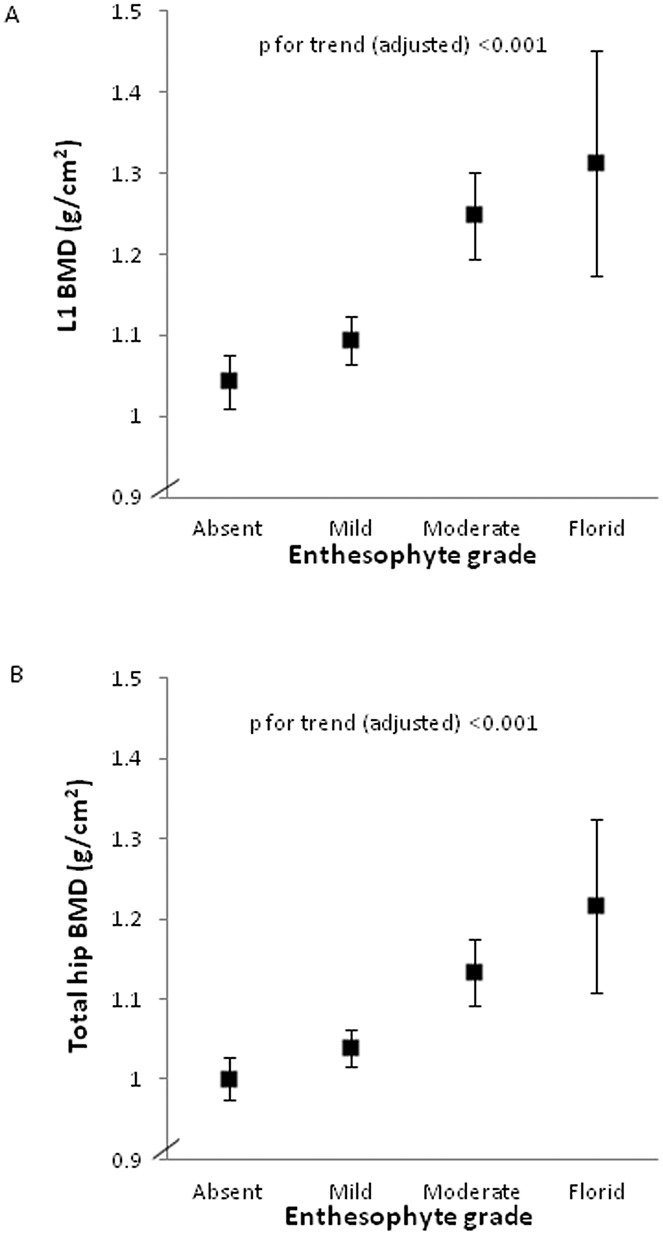
Bone mineral density (BMD) at the L1 lumbar vertebra (A) and at the total hip (B) according to enthesophyte grade in the combined population of high bone mass (HBM) cases and pooled controls. Bars show the mean and 95% confidence interval. When the BMD values for both the right hip and the left hip were available (n = 73), the mean value was used. BMD variables were standardized according to the scanner type (Hologic for the Chingford and Hertfordshire study controls, mixed Lunar/Hologic for the HBM cases and family controls). *P* values for trend were determined in analyses adjusted for age, sex, and body mass index. The BMD values at L1 were approximately normally distributed, while the BMD values at the total hip had 1 extreme outlying value (exclusion of which did not materially change the results). In A, n = 217 HBM cases and n = 426 pooled controls. In B, n = 218 HBM cases and n = 423 pooled controls.

To establish whether a dose-response relationship exists between BMD and the presence of enthesophytes, we performed an analysis of BMD according to enthesophyte grade in the combined population of cases and controls. A trend toward increasing BMD (unadjusted mean values) at the L1 vertebra and at the total hip was observed with increasing enthesophyte grade (Figures 3A and B). This association persisted at both sites after full adjustment for age, sex, and BMI (*P* for trend < 0.001). Interestingly, when we stratified these analyses by HBM case or control status, we found that the associations between BMD and enthesophyte grade were mainly driven by the control group (results not shown), with a significant interaction by case–control status seen at the total hip (interaction *P* = 0.01) but not at L1 (interaction *P* = 0.4).

#### Enthesophytes versus osteophytes

**Figure 4:**
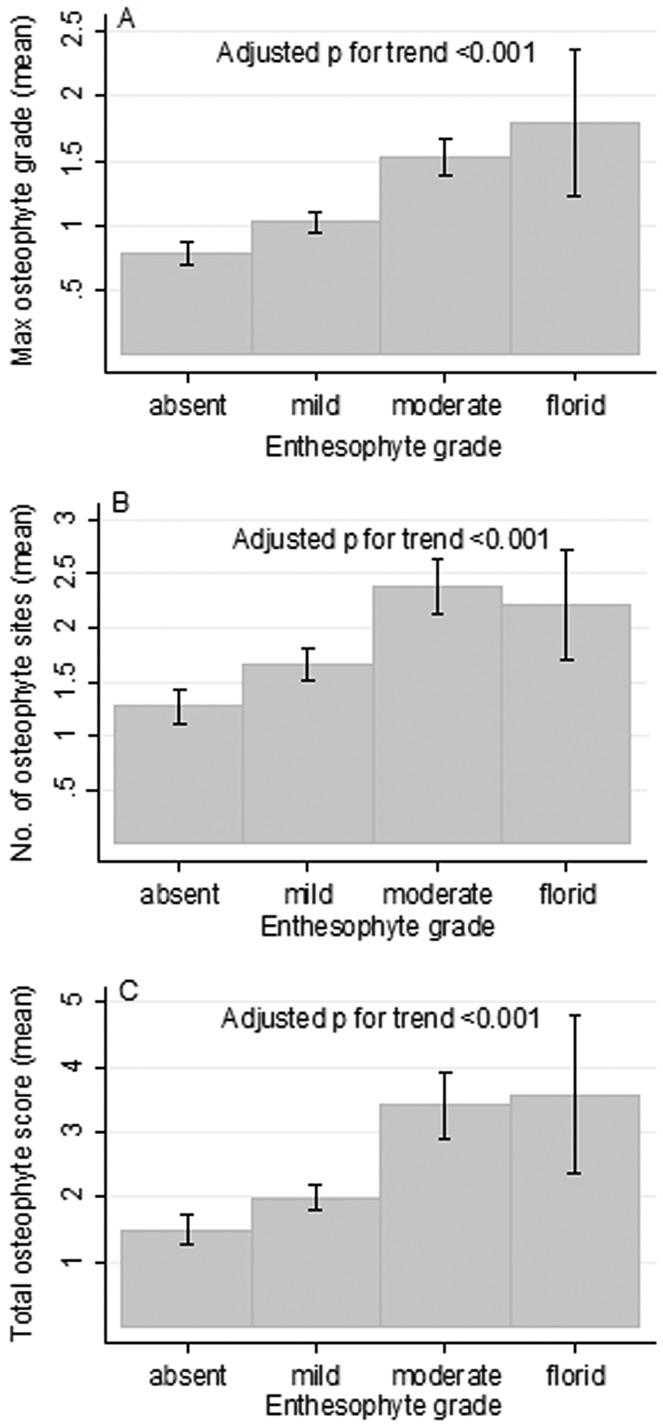
Mean maximum osteophyte grade per pelvis (scale 0–3) (A), mean number of osteophyte sites per pelvis (scale 0–6) (B), and mean total osteophyte score (derived by summing the grades of all osteophytes on radiographs; scale 0–18) (C) according to enthesophyte grade (absent, mild, moderate, or florid) in the combined population of high bone mass cases and controls. Bars show the mean and 95% confidence interval (absent n = 241, mild n = 311, moderate n = 97, florid n = 14). *P* values for trend were determined in analyses adjusted for age, sex, and body mass index.

Having established an association between HBM case status and the presence of both enthesophytes and osteophytes, we next investigated whether enthesophytes and osteophytes were associated irrespective of case status. As shown in Figure 4A, in the study population overall, the enthesophyte grade was positively associated with the maximum osteophyte grade. Positive associations were also observed between the enthesophyte grade and the number of osteophyte sites per pelvis (Figure 4B), as well as between the enthesophyte grade and the total osteophyte score (Figure 4C).

Regression analyses were performed to examine the associations between enthesophytes and osteophytes, in both unadjusted analyses and analyses fully adjusted for age, sex, and BMI (results available from the corresponding author upon request). The fully adjusted OR for the presence (versus absence) of any enthesophyte per unit increase in the highest osteophyte grade was 1.88 (95% CI 1.46–2.43, *P* < 0.001). Similarly, there was a 3-fold increase in the odds of observing moderate enthesophytes per unit increase in osteophyte grade (OR 2.99 [95% CI 2.19–4.07], *P* < 0.001). For each unit increase in enthesophyte grade, the odds of any osteophyte being present were increased >2-fold (OR 2.31 [95% CI 1.68–3.16], *P* < 0.001). Similarly, the odds of observing any moderate osteophyte per unit increase in enthesophyte grade were increased >2-fold (OR 2.49 [95% CI 1.89–3.29], *P* < 0.001).

These associations were then examined separately in HBM cases and controls to investigate whether the relationship between enthesophytes and osteophytes differed according to HBM status. Although point estimates were greater in controls compared with cases, the interaction *P* values were all >0.1, suggesting that these associations were similar in the 2 groups (results available from the corresponding author upon request).

## DISCUSSION

To our knowledge, this is the first study to evaluate the presence of radiographic enthesophytes in a population of individuals with extremely high bone mass. Consistent with our prior hypothesis, we observed a higher prevalence of pelvic enthesophytes among HBM cases compared with controls. Moreover, BMD values in both the hip and lumbar spine, as assessed by DXA, increased with increasing severity of enthesophytes. Furthermore, we confirmed the observation made in archaeologic studies of an association between the presence of enthesophytes and the presence of osteophytes. Other investigators previously labeled individuals with this combination of features as “bone-formers” (3).

It has been reported that individuals with DISH may have increased BMD at several sites, including the distal radius and lumbar spine/hip (24,25). This finding, rather than representing a true increase in BMD, could be attributed to ossified ligaments within the DXA field, leading to artefactual increases in measured BMD (21). We therefore performed a sensitivity analysis that excluded individuals whose pelvic radiographs showed moderate or florid enthesophytes and who also had evidence of DISH-like changes affecting the L1 vertebra (used to define HBM case status). The overall association between HBM and enthesophytes, although slightly attenuated, remained robust, suggesting that artefactually increased lumbar BMD measurements due to DISH do not explain our findings in the majority of cases. Similarly, the positive association we observed between hip BMD and enthesophyte grade could not be explained by the presence of DISH.

OA features such as osteophytes and subchondral sclerosis within the DXA field could potentially lead to misclassification of HBM case status. Evidence from the published literature suggests that whereas lumbar spine OA contributes to artefactual elevation of the BMD, hip OA has only a minimal influence on the measured hip BMD on DXA (26). For this reason, the L1 vertebra was included in our definition of HBM, since the L1 Z score was not associated with the severity of lumbar OA previously assessed on DXA images (16). In addition, the fact that strong associations between HBM and both enthesophytes and osteophytes persisted in analyses restricted to the group of HBM cases defined by high BMD at the hip suggests that misclassification due to the presence of lumbar spine OA does not explain our findings.

Osteophytes and enthesophytes share several common features. For example, endochondral ossification has been shown to be involved in the formation of osteophytes and some enthesophytes (5,27,28). Furthermore, although the precise triggers for the formation of osteophytes and enthesophytes remain unclear, mechanical stimuli are likely to play a role (27–29). Animal models have shown that osteophyte formation may be induced by altered joint mechanics (e.g., following destabilization of the medial meniscus [30]), and osteophyte formation at specific sites within human knee joints has been related to biomechanical factors such as varus malalignment (31). Similarly, observations indicating that enthesophytes develop in the direction of pull of the relevant tendon or ligament (3) and may develop in response to repetitive strain (3,5) support a role for mechanical stress in the formation of enthesophytes.

Theoretically, the observed association between HBM and osteophytosis could have arisen either from altered joint loading secondary to increased bone mass or as a result of an increased bone-formation response to a given load. However, in the case of enthesophytes, altered bone mechanoresponsiveness, rather than altered bone loading per se, is presumably responsible. Taken together, these findings suggest that altered bone responsiveness may underlie the apparent triad of osteophytosis, enthesophyte formation, and increased bone mass that we observed. HBM is associated not only with an increased risk of osteophytosis, but also with clinical end points related to OA, such as hip replacement (32). Therefore, it is tempting to speculate that the tendency toward excess bone formation associated with this triad contributes to the pathogenesis of certain subtypes of OA.

Plausible biologic explanations for the proposed increased bony proliferative response in these individuals include alterations in growth factor expression (27), which are presumably related to genetic factors (3). Although the genetic basis for HBM in the majority of our cases is unknown and is the subject of ongoing studies, a genome-wide association study in this HBM population has shown overrepresentation of single-nucleotide polymorphisms known to be associated with BMD in the wider population, including loci in Wnt pathway/endochondral ossification genes (33).

The canonical Wnt signaling pathway is known to play a key role in the osteoblast response of bone to mechanical loading (34), and genetic mutations activating this pathway result in an HBM phenotype (35). Increased osteogenic activity, arising from up-regulation of Wnt signaling, could theoretically lead to both increased BMD and a propensity to form enthesophytes/osteophytes in response to normal or abnormal mechanical strains (analogous to the increased osteogenic responsiveness seen in mice heterozygous for *LRP5*-activating mutations [36]). Interestingly, reduced levels of Dkk-1 (a Wnt pathway inhibitor) have been associated with the radiographic severity of both DISH (37) and knee OA (38,39), and polymorphisms within the Wnt pathway have also been linked to the risk of OA in genetic studies (40,41).

This study focused on a population with the rare HBM phenotype. It is possible that OA in this group may not be representative of OA in the general population. However, given the BMD–enthesophyte and osteophyte–enthesophyte associations observed when cases and controls were combined, it is tempting to speculate that these relationships may hold true for OA more generally, or at least for certain subtypes of the disease. One proposed phenotypic classification of OA is based on the local bony response, distinguishing “hypertrophic” OA, characterized by osteophytes/sclerosis, from “atrophic” forms of the disease, lacking bony features (42–44). Our study suggests that the presence of radiographic enthesophytes, in addition to osteophytes, might help to define a subtype of OA in which a bone-formation response predominates. Alternatively, it has been proposed that systemic enthesopathy-related OA should be considered as a specific subphenotype in its own right, since it has been postulated that in some cases, ligament/tendon changes may play a primary role in initiating the OA process (45).

Our study has several limitations. We did not attempt to examine associations between enthesophytes and clinical symptoms such as hip pain, or other radiographic OA features such as joint space narrowing. Our methods for grading radiographic pelvic enthesophytes were subjective, because of the absence of any established grading method (although there have been 2 previous small studies in which a semiquantitative scoring system was used [46,47]). The fact that osteophytes and enthesophytes were graded by a single observer in one sitting raises the possibility that the presence of osteophytes may have prompted a more thorough search for enthesophytes, and vice versa. Our method has also not been validated against direct examination of skeletons, arguably the most definitive method for assessing enthesophytes (4); a radiographic approach is likely to be less sensitive.

Another limitation is the relatively high number of incomplete control radiographs that were excluded. However, because the controls excluded for this reason tended to be younger and more often female, this would, if anything, bias our results toward the null by increasing the prevalence of enthesophytes within the control group. Another issue (in common with direct examination of skeletons [27]) is that some osteophytes, particularly around the acetabulum, might have been more accurately termed enthesophytes.

Finally, some additional potential confounders, including smoking, past and present use of steroids, and alcohol intake, were available at the time of the radiographic evaluations in the HBM cases and family controls only, and therefore could not be adjusted for in the main analysis. However, adjusting for these variables in analyses restricted to the HBM study population did not attenuate the associations between HBM, enthesophytes, and osteophytes. Another potential confounder was physical activity, for which data were not available in a consistent format across studies.

In conclusion, our results have demonstrated an increased prevalence of radiographic pelvic enthesophytes in a population of individuals with extreme HBM. This group is also known to have an increased prevalence of radiographic hip OA, characterized by osteophytes, and we have further shown that the presence of osteophytes and the presence of enthesophytes is associated in these individuals. We speculate that the triad of osteophytosis, enthesophyte formation, and increased bone mass may identify a subtype of OA primarily caused by increased bone formation. Enthesophytes might be usefully added to existing definitions of hypertrophic OA in order to identify this particular phenotype. It is hoped that exome sequencing of this unique HBM population, which is currently under way, may provide new insights into the molecular mechanisms regulating these processes.

## AUTHOR CONTRIBUTIONS

All authors were involved in drafting the article or revising it critically for important intellectual content, and all authors approved the final version to be published. Dr. Hardcastle had full access to all of the data in the study and takes responsibility for the integrity of the data and the accuracy of the data analysis.

**Study conception and design.** Hardcastle, Dieppe, Gregson, Spector, Cooper, Williams, Davey Smith, Tobias.

**Acquisition of data.** Hardcastle, Dieppe, Gregson, Arden, Spector, Hart, Edwards, Dennison, Cooper, Williams, Tobias.

**Analysis and interpretation of data**. Hardcastle, Dieppe, Gregson, Arden, Spector, Edwards, Cooper, Davey Smith, Tobias.

## References

[b1] Resnick D, Niwayama G (1983). Entheses and enthesopathy: anatomical, pathological, and radiological correlation. Radiology.

[b2] Slobodin G, Rozenbaum M, Boulman N, Rosner I (2007). Varied presentations of enthesopathy. Semin Arthritis Rheum.

[b3] Rogers J, Shepstone L, Dieppe P (1997). Bone formers: osteophyte and enthesophyte formation are positively associated. Ann Rheum Dis.

[b4] Shaibani A, Workman R, Rothschild BM (1993). The significance of enthesopathy as a skeletal phenomenon. Clin Exp Rheumatol.

[b5] Benjamin M, Rufai A, Ralphs JR (2000). The mechanism of formation of bony spurs (enthesophytes) in the Achilles tendon. Arthritis Rheum.

[b6] Rogers J, Shepstone L, Dieppe P (2004). Is osteoarthritis a systemic disorder of bone?. Arthritis Rheum.

[b7] Kalichman L, Malkin I, Kobyliansky E (2007). Hand bone midshaft enthesophytes: the influence of age, sex, and heritability. Osteoarthritis Cartilage.

[b8] Gibson N, Guermazi A, Clancy M, Niu J, Grayson P, Aliabadi P (2012). Relation of hand enthesophytes with knee enthesopathy: is osteoarthritis related to a systemic enthesopathy?. J Rheumatol.

[b9] Tan AL, Grainger AJ, Tanner SF, Shelley DM, Pease C, Emery P (2005). High-resolution magnetic resonance imaging for the assessment of hand osteoarthritis. Arthritis Rheum.

[b10] Hart DJ, Mootoosamy I, Doyle DV, Spector TD (1994). The relationship between osteoarthritis and osteoporosis in the general population: the Chingford Study. Ann Rheum Dis.

[b11] Dequeker J, Aerssens J, Luyten FP (2003). Osteoarthritis and osteoporosis: clinical and research evidence of inverse relationship. Aging Clin Exp Res.

[b12] Hannan MT, Anderson JJ, Zhang Y, Levy D, Felson DT (1993). Bone mineral density and knee osteoarthritis in elderly men and women: the Framingham Study. Arthritis Rheum.

[b13] Nevitt MC, Lane NE, Scott JC, Hochberg MC, Pressman AR, Genant HK, Study of Osteoporotic Fractures Research Group (1995). Radiographic osteoarthritis of the hip and bone mineral density. Arthritis Rheum.

[b14] Hardcastle SA, Dieppe P, Gregson CL, Hunter D, Thomas G, Arden NK (2014). Prevalence of radiographic hip osteoarthritis is increased in high bone mass. Osteoarthritis Cartilage.

[b15] Gregson CL, Steel SA, O'Rourke KP, Allan K, Ayuk J, Bhalla A (2012). ‘Sink or swim’: an evaluation of the clinical characteristics of individuals with high bone mass. Osteoporos Int.

[b16] Gregson CL, Steel SA, Yoshida K, Reid D, Tobias JH

[b17] http://www.wma.net/en/30publications/10policies/b3/17c.pdf.

[b18] Syddall HE, Aihie Sayer A, Dennison EM, Martin HJ, Barker DJ, Cooper C (2005). Cohort profile: the Hertfordshire cohort study. Int J Epidemiol.

[b19] Schaap LA, Peeters GM, Dennison EM, Zambon S, Nikolaus T, Sanchez-Martinez M (2011). European Project on OSteoArthritis (EPOSA): methodological challenges in harmonization of existing data from five European population-based cohorts on aging. BMC Musculoskelet Disord.

[b20] Burnett S, Hart DJ, Cooper C, Spector TD (1994). A radiographic atlas of osteoarthritis.

[b21] Westerveld LA, Verlaan JJ, Lam MG, Scholten WP, Bleys RL, Dhert WJ (2009). The influence of diffuse idiopathic skeletal hyperostosis on bone mineral density measurements of the spine. Rheumatology (Oxford).

[b22] Diederichs G, Engelken F, Marshall LM, Peters K, Black DM, Issever AS (2011). Diffuse idiopathic skeletal hyperostosis (DISH): relation to vertebral fractures and bone density. Osteoporos Int.

[b23] Resnick D, Niwayama G (1976). Radiographic and pathologic features of spinal involvement in diffuse idiopathic skeletal hyperostosis (DISH). Radiology.

[b24] Di Franco M, Mauceri MT, Sili-Scavalli A, Iagnocco A, Ciocci A (2000). Study of peripheral bone mineral density in patients with diffuse idiopathic skeletal hyperostosis. Clin Rheumatol.

[b25] Sahin G, Polat G, Bagis S, Milcan A, Erdogan C (2002). Study of axial bone mineral density in postmenopausal women with diffuse idiopathic skeletal hyperostosis related to type 2 diabetes mellitus. J Women's Health (Larchmt).

[b26] Liu G, Peacock M, Eilam O, Dorulla G, Braunstein E, Johnston CC (1997). Effect of osteoarthritis in the lumbar spine and hip on bone mineral density and diagnosis of osteoporosis in elderly men and women. Osteoporos Int.

[b27] Felson DT, Neogi T (2004). Osteoarthritis: is it a disease of cartilage or of bone?. Arthritis Rheum.

[b28] Van der Kraan PM, van den Berg WB (2007). Osteophytes: relevance and biology. Osteoarthritis Cartilage.

[b29] Goldring SR (2009). Role of bone in osteoarthritis pathogenesis. Med Clin North Am.

[b30] Loeser RF, Olex AL, McNulty MA, Carlson CS, Callahan M, Ferguson C (2013). Disease progression and phasic changes in gene expression in a mouse model of osteoarthritis. PloS One.

[b31] Nagaosa Y, Lanyon P, Doherty M (2002). Characterisation of size and direction of osteophyte in knee osteoarthritis: a radiographic study. Ann Rheum Dis.

[b32] Hardcastle SA, Gregson CL, Deere KC, Davey Smith G, Dieppe P, Tobias JH (2013). High bone mass is associated with an increased prevalence of joint replacement: a case–control study. Rheumatology (Oxford).

[b33] Gregson C, Leo PJ, Clark GR, Davey Smith G, Brown MA, Tobias JH (2013). A GWAS in an extreme high bone mass population shows excess signal from genes associated with BMD in the normal population. Bone Abstracts 2013.

[b34] Bonewald LF, Johnson ML (2008). Osteocytes, mechanosensing and Wnt signaling. Bone.

[b35] Little RD, Carulli JP, Del Mastro RG, Dupuis J, Osborne M, Folz C (2002). A mutation in the LDL receptor-related protein 5 gene results in the autosomal dominant high-bone-mass trait. Am J Hum Genet.

[b36] Saxon LK, Jackson BF, Sugiyama T, Lanyon LE, Price JS (2011). Analysis of multiple bone responses to graded strains above functional levels, and to disuse, in mice in vivo show that the human Lrp5 G171V High Bone Mass mutation increases the osteogenic response to loading but that lack of Lrp5 activity reduces it. Bone.

[b37] Senolt L, Hulejova H, Krystufkova O, Forejtova S, Andres Cerezo L, Gatterova J (2012). Low circulating Dickkopf-1 and its link with severity of spinal involvement in diffuse idiopathic skeletal hyperostosis. Ann Rheum Dis.

[b38] Honsawek S, Tanavalee A, Yuktanandana P, Ngarmukos S, Saetan N, Tantavisut S (2010). Dickkopf-1 (Dkk-1) in plasma and synovial fluid is inversely correlated with radiographic severity of knee osteoarthritis patients. BMC Musculoskelet Disord.

[b39] Voorzanger-Rousselot N, Ben-Tabassi NC, Garnero P (2009). Opposite relationships between circulating Dkk-1 and cartilage breakdown in patients with rheumatoid arthritis and knee osteoarthritis. Ann Rheum Dis.

[b40] Castano Betancourt MC, Cailotto F, Kerkhof HJ, Cornelis FM, Doherty SA, Hart DJ (2012). Genome-wide association and functional studies identify the DOT1L gene to be involved in cartilage thickness and hip osteoarthritis. Proc Natl Acad Sci U S A.

[b41] Baker-Lepain JC, Lynch JA, Parimi N, McCulloch CE, Nevitt MC, Corr M (2012). Variant alleles of the Wnt antagonist FRZB are determinants of hip shape and modify the relationship between hip shape and osteoarthritis. Arthritis Rheum.

[b42] Solomon L (1976). Patterns of osteoarthritis of the hip. J Bone Joint Surg Br.

[b43] Ledingham J, Dawson S, Preston B, Milligan G, Doherty M (1992). Radiographic patterns and associations of osteoarthritis of the hip. Ann Rheum Dis.

[b44] Javaid MK, Lane NE, Mackey DC, Lui LY, Arden NK, Beck TJ (2009). Changes in proximal femoral mineral geometry precede the onset of radiographic hip osteoarthritis: the study of osteoporotic fractures. Arthritis Rheum.

[b45] McGonagle D, Tan AL, Carey J, Benjamin M (2010). The anatomical basis for a novel classification of osteoarthritis and allied disorders. J Anat.

[b46] Fahrer H, Barandum R, Gerber NJ, Friederich NF, Burkhardt B, Weisman MH (1989). Pelvic manifestations of diffuse idiopathic skeletal hyperostosis (DISH): are they clinically relevant?. Rheumatol Int.

[b47] Haller J, Resnick D, Miller CW, Schils JP, Kerr R, Bielecki D (1989). Diffuse idiopathic skeletal hyperostosis: diagnostic significance of radiographic abnormalities of the pelvis. Radiology.

[b48] Hui SL, Gao S, Zhou XH, Johnston CC, Lu Y, Gluer CC (1997). Universal standardization of bone density measurements: a method with optimal properties for calibration among several instruments. J Bone Miner Res.

[b49] Hanson J (1997). Standardization of femur BMD. J Bone Miner Res.

